# Demonstration and Characterization of a Persistent Pheromone Lure for the Navel Orangeworm, *Amyelois transitella* (Lepidoptera: Pyralidae)

**DOI:** 10.3390/insects5030596

**Published:** 2014-07-22

**Authors:** Bradley S. Higbee, Charles S. Burks, Thomas E. Larsen

**Affiliations:** 1 Paramount Farming, Inc., 6801 E Lerdo Highway, Shafter, CA 93263, USA; E-Mail: bradh@paramountfarming.com; 2 USDA, Agricultural Research Service, San Joaquin Valley Agricultural Sciences Center, 9611 South Riverbend Avenue, Parlier, CA 93648, USA; 3 Suterra LLC, 20950 NE Talus Pl, Bend, OR 97701, USA; E-Mail: TomL@amvac-chemical.com

**Keywords:** *Amyelois transitella*, navel orangeworm, pheromone lure, almond, *Prunus dulcis*, pistachio, *Pistacia vera*

## Abstract

The lack of an effective pheromone lure has made it difficult to monitor and manage the navel orangeworm, *Amyelois transitella* (Lepidoptera: Pyralidae), in the economically important crops in which it is the primary insect pest. A series of experiments was conducted to demonstrate and characterize a practical synthetic pheromone lure for capturing navel orangeworm males. Traps baited with lures prepared with 1 or 2 mg of a three- or four-component formulation captured similar numbers of males. The fluctuation over time in the number of males captured in traps baited with the pheromone lure correlated significantly with males captured in female-baited traps. Traps baited with the pheromone lure usually did not capture as many males as traps baited with unmated females, and the ratio of males trapped with pheromone to males trapped with females varied between crops and with abundance. The pheromone lure described improves the ability of pest managers to detect and monitor navel orangeworm efficiently and may improve management and decrease insecticide treatments applied as a precaution against damage. Awareness of differences between male interaction with the pheromone lure and calling females, as shown in these data, will be important as further studies and experience determine how best to use this lure for pest management.

## 1. Introduction

The navel orangeworm, *Amyelois transitella* (Walker) (Lepidoptera: Pyralidae), is the principal insect pest of almonds and pistachios and an important pest of walnuts. In aggregate, these crops are planted on >460,000 million ha in CA and generate >$5 billion yearly in their unprocessed form [1]. Pest management for the navel orangeworm is affected by ways in which its biology differs from other widely studied orchard pests, such as the codling moth, *Cydia pomonella* (L.) [2], and the oriental fruit moth, *Grapholita molesta* (Busck) [3]. Compared to these moths, the navel orangeworm is more polyphagous [4]. It is less tied to particular hosts and more to the state of maturity or senescence of the host [4]. Host quality and developmental rate are thus more variable compared to other moth pests [5,6,7]. Pistachios support larger navel orangeworm populations than almonds when both crops receive similarly rigorous sanitation efforts [8].

The lack of an effective pheromone lure has hindered integrated management of the navel orangeworm. In other important lepidopteran pests of horticultural tree crops, monitoring with pheromone lures is an important part of the overall management strategy [9]. Up to the present, egg traps have been used for monitoring the navel orangeworm [10,11,12,13,14,15]. Egg traps have several disadvantages compared to pheromone traps. After hull split in almonds, they detect less navel orangeworm activity compared to trapping with black lights or with unmated females as a pheromone source [16]. In pistachios, this apparent loss of sensitivity of egg traps following the first overwintering flight is even more striking [17]. Egg traps are labor-intensive compared to pheromone traps, and an over-dispersed distribution of the eggs on traps means that pest managers frequently do not have sufficient egg traps within a block to determine local trends with sufficient confidence [14]. Monitoring with unmated females as a pheromone source has provided useful research data [15,16], but is not sufficiently economical or robust for commercial use. An effective sex pheromone lure for this species has thus long been sought.

The odd and complex nature of the sex pheromone in the navel orangeworm has doubtlessly impeded the development of such a pheromone lure. A major component, (11*Z*,13*Z*)-hexadecadienal, was discovered in 1979 [18]. In the field, however, traps baited with this compound by itself captured far fewer males than traps baited with unmated females [19]. This is in contrast to some other economically important species in which a single major component of the pheromone blend is, by itself, at least sufficient to trap males in the field [20,21]. Subsequent studies found additional components associated with the sex pheromone of this species [19,22] and determined that a blend of (11*Z*,13*Z*)-hexadecadienal, (11*Z*,13*Z*)-hexadecadien-1-ol, (11*Z*,13*E*)-hexadecadien-1-ol and (3*Z*,6*Z*,9*Z*,12*Z*,15*Z*)-tricosapentaene is necessary and sufficient for optimal attraction [23]. Despite this improved knowledge of the sex pheromone, lures placed in the field often lost attractiveness after a single night. The instability of the conjugated dienes and differential release rates of the 16- and 23-carbon components may have hindered the development of a persistent lure in the field, even after an attractive blend was identified.

Here, we present experiments to demonstrate and characterize a sex pheromone lure for the navel orangeworm. An initial experiment in 2011 compared males captured with the newly-developed sex pheromone lure in almonds over a 64-day period. Experiments in 2012 examined different blend compositions and pheromone loads in almonds and pistachios. This experiment was repeated with a greater distance between traps. Based on these data, we discuss the effects of crop and the abundance of navel orangeworm on the performance of this sex pheromone lure.

## 2. Experimental

### 2.1. Insects, Traps and Test Sites

Orange wing traps (Suterra LLC, Bend OR), modified as described in [24], and baited with three one- or two-night-old unmated females sealed in a mesh bag, as described in [25], were used as a standard for the comparison of the performance of synthetic lures. A previous study showed that, under mid-summer conditions, females prepared in this manner survived and attracted males for at least 4 nights [26]. Females were reared from a laboratory strain maintained at the Paramount Farming Company Belridge Laboratory (Lost Hills, CA, USA). This laboratory strain, from the USDA-ARS in Parlier, CA, was founded from almonds and received from the University of California, Berkeley, in 1966. At the Belridge laboratory, this strain was maintained on a wheat bran diet [27] at 25 °C with a 16:8 L:D photoperiod. The strain was also infused periodically with feral adults emerged from infested almond and pistachio harvest samples. Traps were hung in trees, 1.5 m above the ground, as near as possible to the stated trap spacings.

The experiments were conducted on almond ranches and pistachio ranches owned by Paramount Farming, LLC, and located in Kern County (Lost Hills, CA, USA) (Table 1). The almond and pistachio orchards were planted at respective densities of 213 and 326 trees per ha. Pistachios are typically planted more densely and replanted less frequently compared to almonds [8].

**Table 1:** Characteristics of experimental sites.

Ranch	Crop	Latitude	Longitude	Age in 2011	Experiment
1	Almond	35.49	−119.69	22	1
2	Almond	35.52	−119.74	12	1, 2
3	Almond	35.49	−119.67	23	2
4	Pistachio	35.62	−119.93	39	2
5	Almond	35.64	−119.98	6	2, 3
6	Pistachio	35.62	−119.96	39	3

### 2.2. Chemicals and Emitters

The standard navel orangeworm pheromone blend for these experiments contained (11*Z*,13*Z*)-hexadecadienal, (11*Z*,13*Z*)-hexadecadien-1-ol, (11*Z*,13*E*)-hexadecadien-1-ol and (3*Z*,6*Z*,9*Z*,12*Z*,15*Z*)-tricosapentaene in a ratio of 45:45:2:7. Each of the pheromone components was >95% purity as measured by gas chromatography. The (11*Z*,13*Z*)-hexadecadienal was manufactured by Suterra LLC (Bend, OR). The (11*Z*,13*Z*)-hexadecadien-1-ol and (11*Z*,13*E*)-hexadecadien-1-ol were manufactured by Bedoukian Research Inc. (Danbury, CT, USA), and tricosapentaene was synthesized by Michael Chong (University of Waterloo, Ontario, Canada). A three-component version of the lure omitted (11*Z*,13*E*)-hexadecadien-1-ol and contained the remaining components in the ratio of 93:93:14. The emitter, a foil-lined reservoir with a plastic release membrane, was a proprietary product from Suterra LLC. A version of this lure, as optimized based on the present research, is currently sold as NOW Biolure.

### 2.3. Specific Experiments

Preliminary experiments in 2011 aided in the selection from among several available proprietary reservoir release devices, carriers and stabilizers. Experiment 1, starting on September 15, 2011, compared males captured in wing traps baited either with this resulting lure or with calling females. The preliminary experiments were conducted in pistachios, because that crop often has a greater abundance of navel orangeworm compared to almonds [8]. This first experiment with the standard lure configuration was, however, conducted in almonds, because navel orangeworm abundance is generally higher in almonds in fall compared to earlier in the year and because almonds are harvested earlier than pistachios, and therefore, harvest activities presented less of a problem. Wing traps baited with the attractants were arranged in a randomized complete block design, with a total of eight replicate blocks (four each at the two sites) 29 m apart, and treatments placed randomly 13 m apart within blocks. Unmated females were changed 15 times, at intervals of 3–4 days. Trap counts were recorded each time the females in the female-baited traps were changed, for a total duration of 64 days.

Two additional experiments examined the effect of the amount of material loaded into synthetic lures and compared a three-component blend with the standard four-component blend. These experiment were conducted simultaneously in both almonds and pistachios, because differences in the response between the first experiment in almonds (described above) and preliminary trials in pistachios (data not shown) suggested that the relative effectiveness of traps baited with either the artificial lure or unmated females might differ between these crops. Two trap densities were also examined, because previous studies have indicated that the apparent difference in attractiveness of semiochemical treatments can be influenced by the distance between them [28].

Reservoir membranes were prepared with one or two mg of the three- or four-component blend, comprising four treatments. Empty traps and traps baited with unmated females comprised two additional treatments, for a total of six. In Experiment 2, wing traps containing these treatments were placed as a randomized complete block arrangement with ten replicate blocks each in almond and pistachio ranches. Blocks were separated by at least 37 m, and traps within blocks were separated by approximately 18 m. Experiment 2 was initiated March 29, 2012. Unmated females were changed 26 times, at intervals of 3 to 4 days. Trap counts were recorded each time the females in the female-baited traps were changed, for a total duration of 91 days.

Experiment 3 examined the same treatments as Experiment 2 and was conducted in ten replicate blocks in almond plantings and nine replicate blocks in pistachios. However, Experiment 3 differed from Experiment 2 in that there were larger minimum distances between blocks (120 m), and the distances between treatments within blocks were 3.3× greater (60 m). Experiment 3 was initiated April 29, 2012. Unmated females were changed 18 times, at intervals of 3 to 4 days. Trap counts were recorded each time the females in the female-baited traps were changed, for a total duration of 64 days.

### 2.4. Statistical Analysis

Data were analyzed using the SAS System [29]. Correlation analysis (PROC CORR) was used to compare temporal trends in the mean males captured between traps baited with pheromone lure preparations and live females. The period examined with correlation was determined by initial examination of the plotted data. For Experiment 1, the Pearson correlation was used, allowing the determination of the coefficient of determination, *r*^2^ [30]. For Experiments 2 and 3, the data for individual monitoring intervals did not support the assumption of a bivariate normal distribution [30], and correlation analysis was done using the nonparametric Spearman rank correlation (ρ).

The cumulative number of males captured was compared between traps baited with females and lure preparations using generalized linear mixed models (including ANOVA). A mixed-model ANOVA (PROC MIXED) was used in Experiment 1. The cumulative males captured was the response variable; the bait (females or pheromone lure) was the fixed predictor variable, and the ranch and block nested in the ranch were random variables. For Experiments 2 and 3, cumulative males captured were compared between the two crops and trap densities. Trap captures were summed over the first 42 days of these experiments, based on plots of trap data, and also because this monitoring interval was common between Experiments 2 and 3. The blank treatment in these experiments was not included in analysis, because very few males were captured [31]. Mixed-model ANOVA was used for almonds, but a generalized linear mixed model (PROC GLIMMIX) with a negative binomial distribution was used for the pistachio data, because it was more suitable to the wide range of response between treatments. The response variable was the cumulative males captured; the fixed predictor variable was the bait (females or pheromone lure preparation), and the ranch and block nested in the ranch were random variables. A replicate nested in the ranch had 0 covariance and was not used. An alpha of 0.05 was used to determine significance, with a Tukey adjustment for multiple comparisons.

## 3. Results and Discussion

**Figure 1:**
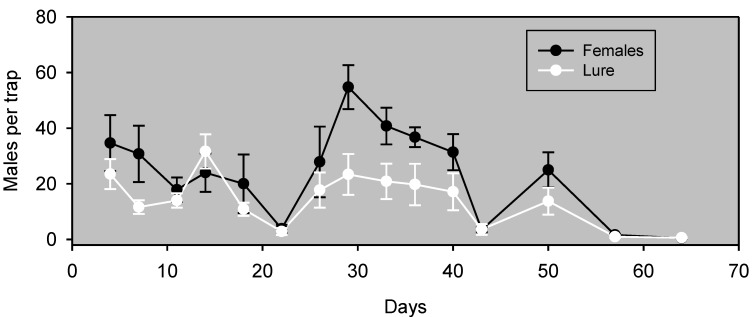
Males captured (mean ± SE, n = 8) in wing traps baited with unmated females *vs*. emitters. Experiment 5 was conducted in almonds and began on September 15, 2011. Blocks were 29 m apart, and traps within blocks were 13 m apart.

Initial experiments (not presented) determined which of several reservoir-type emitters to use, and identified a four-component blend as a standard for comparison. Experiment 1 examined the field persistence of this lure by comparing, in almonds, males captured in traps baited with this pheromone lure *vs*. unmated females. Traps baited with females captured significantly more males (*F*_1,7_ = 5.76, *p* = 0.0475) than traps baited with emitters (353 ± 44 and 212 ± 47 males/trap, respectively; n = 8). The synthetic lures nonetheless were attractive and correlated well with males captured in female-baited traps (*r*^2^ = 0.65, *p* = 0.0003) for >50 days (Figure 1). This persistence represents a substantial improvement over the one or two nights of useful field life obtained with pheromone release devices for navel orangeworm.

**Figure 2:**
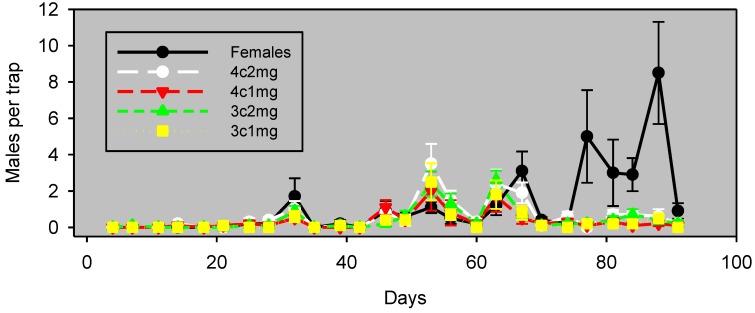
Experiment 2: males captured (mean ± SE) in almonds, by monitoring the interval in wing traps baited with unmated females or artificial lures, starting March 29, 2012. Blocks were 37 m apart, and traps within blocks were 18 m apart. The lure preparation is as in Table 2: 4c2mg, 2 mg of the four-component formulation; 4c1mg, 1 mg of the four-component formulation; 3c2mg, 2 mg of the three-component formulation; and 3c1mg, 1 mg of the three-component formulation.

Experiments 2 and 3 compared four lure preparations (1 or 2 mg of three- or four-component formulations) using different trap densities in almonds and pistachios. In Experiment 2 in almonds, the males captured in traps baited with pheromone lure preparations track well with males captured in pheromone-baited traps for the first 63 days (Figure 2). During this period, the Spearman correlation with female-baited traps was significant for all lure preparations (Table 2), with a value of ρ ranging from 0.81 to 0.94.

**Table 2:** Correlation (Spearman ρ, *p*) of males captured in lure-baited traps with males captured in traps baited with calling females with traps spaced either narrowly (7 m) or widely (60 m) in almonds or pistachios.

Lure preparation	Narrow spacing		Wide spacing	
	Almond (18)	Pistachio (11)	Almond (15)	Pistachio (15)
4c2mg	0.94, <0.0001	0.74, 0.0093	0.34, 0.2087	0.62, 0.0132
4c1mg	0.87, <0.0001	0.68, 0.0225	0.18, 0.5259	0.52, 0.0493
3c2mg	0.93, <0.0001	0.76, 0.0062	0.41, 0.1329	0.76, 0.0010
3c1mg	0.81, <0.0001	0.61, 0.0454	0.33, 0.2265	0.65, 0.0090

The number of intervals used in the correlation is indicated in parentheses. Lure preparation: 4c2mg, 2 mg of the four-component formulation; 4c1mg, 1 mg of the four-component formulation; 3c2mg, 2 mg of the three-component formulation; and 3c1mg, 1 mg of the three-component formulation.

**Figure 3:**
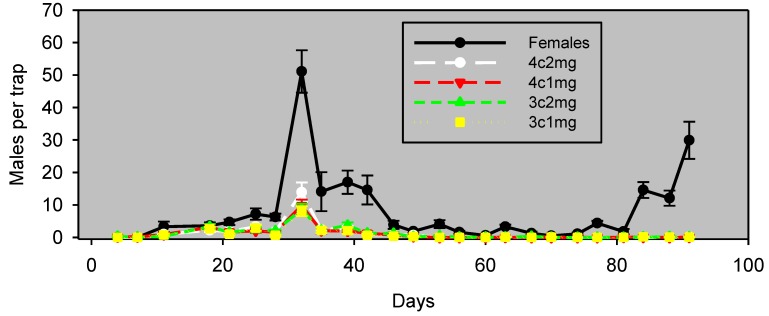
Experiment 2: males captured (mean ± SE) in pistachios, by monitoring the interval in wing traps baited with unmated females or artificial lures, starting March 29, 2012. Blocks were 37 m apart, and traps within blocks were 18 m apart. The lure preparation is as in Table 2.

In Experiment 2 in pistachios (conducted simultaneously), there was a greater difference between males captured with live females *vs*. with pheromone lures, and lure effectiveness was evident only for the first 42 days (Figure 3). During this 42-day period, males captured with each of the lure preparations correlated significantly (*p* < 0.05) with those captured with females, albeit with lower values of ρ (Table 2).

**Figure 4:**
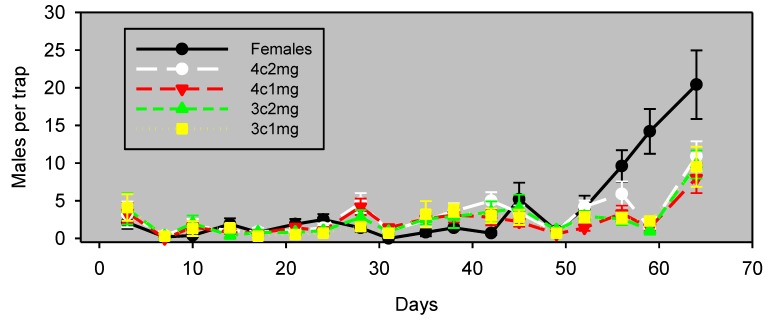
Experiment 3: males captured (mean ± SE) in almonds, by monitoring the interval in wing traps baited with unmated females or artificial lures, starting April 29, 2012. Blocks were 120 m apart, and traps within blocks were 60 m apart. The lure preparation is as in Table 2.

Experiment 3, begun in both almonds and pistachios a month later than Experiment 2, used a greater inter-trap distance to examine whether trap spacing had an impact on the results of these experiments. Almonds in Experiment 3 had a generally low abundance of navel orangeworm, as was the case for Experiment 2 (Figure 4). The number of males captured with pheromone lure generally appeared to track with the number of males trapped with females for the first 52 days, but not thereafter. However, the Spearman coefficient for the correlation of lures and females was not significant during this 52-day period for any of the lure combinations (Table 2).

**Figure 5:**
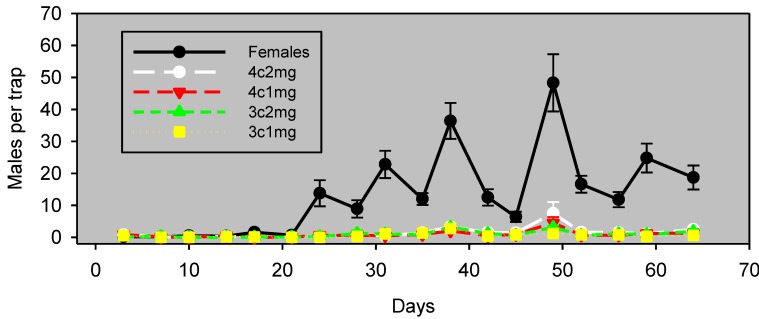
Experiment 3: males captured (mean ± SE) in pistachios, by monitoring the interval in wing traps baited with unmated females or artificial lures, starting April 29, 2012. Blocks were 120 m apart, and traps within blocks were 60 m apart. The lure preparation is as in Table 2.

In pistachios in Experiment 3, many males were captured in female-baited traps, like the previous Experiment 2 and in contrast to the simultaneous trial in almonds. Furthermore, in contrast to almonds, the fluctuations of males captured in traps baited with pheromone lures correlated significantly with those of female-baited traps, even while the number of males captured in female-baited traps greatly outnumbers the number of males captured in traps baited with pheromone lures (Figure 5, Table 2).

Summaries of the simultaneous trials in almonds and pistachios (Experiments 2 and 3) allow the comparison of the four pheromone preparations used in the lures (Table 2 and Table 3). While the performance of these four lure preparations was generally similar, correlation analysis suggested that lure load was more important than composition (three *vs.* four components). This trend was particularly evident in Experiment 2. There were no significant correlations in almonds in Experiment 3, and in pistachios in Experiment 3, the correlation was greater for the three-component lures than for the four-component lures with an equivalent load (Table 2). However, an examination of cumulative males (Table 3) captured suggests that the four-component, 2-mg lure was more effective than the others. While there are no statistically significant differences in males captured with any of the lure preparations in any of the four trials (Experiments 2 and 3 in almonds and pistachios), the number of males in the four-component, 2-mg lure was persistently greater than the others in all four trials. Furthermore, in Experiment 3 in almonds, the four-component, 2-mg lures captured significantly more males than female-baited traps, whereas this was not true for the other three lure preparations. Thus, while there are not clearly significant differences among the four pheromone lure preparations, there is reason to recommend that the four-component, 2-mg lure remain a standard.

**Table 3:** Males per trap (mean ± SE) over the first 42 days of experiments with traps spaced either narrowly (37 m) or widely (60 m).

Crop	Treatment	Narrow	% of Female-baited	Wide	% of Female-baited
Almond	Females	2.3 ± 1.46		14 ± 3.1 ^a^	
	4 component, 2 mg	2.0 ± 0.54	87	26 ± 6.1 ^b^	186
	4 component, 1 mg	1.0 ± 0.21	43	23 ± 4.6 ^ab^	164
	3 component, 2 mg	1.3 ± 0.42	57	22 ± 5.7 ^ab^	157
	3 component, 1 mg	0.8 ± 0.39	35	21 ± 6.0 ^ab^	150
		*F*_4,36_ = 0.75, *p* = 0.57		*F*_4,37_ = 3.07, *p* = 0.0283	
Pistachio	Females	122 ± 18 ^a^		113 ± 16.0 ^a^	
	4 component, 2 mg	30 ± 5.2 ^b^	25	9.3 ± 1.8 ^b^	8
	4 component, 1 mg	25 ± 3.4 ^b^	20	6.6 ± 1.0 ^b^	6
	3 component, 2 mg	26 ± 4.0 ^b^	21	8.6 ± 1.7 ^b^	7
	3 component, 1 mg	21 ± 4.0 ^b^	17	6.8 ± 2.2 ^b^	6
		*F*_4,36_ = 29.98, *p* < 0.0001		*F*_4,32_ = 58.70, *p* < 0.0001	

While the data from the female-baited traps in Experiments 2 and 3 were consistent with previous studies indicating greater abundance in pistachios [8,32], the data from the pheromone lures were not. In Experiment 3, for example, the data from female-baited traps indicated greater abundance in pistachios than in almonds, while the data from the traps baited with the pheromone lure suggest the converse (Table 3). The reason for this difference between crops in the ratio of males captured with pheromone lures *vs*. with females is not clear. The more widely-spaced treatments were examined based on a previous study indicating that placing semiochemical treatments of different attractiveness too closely together can distort results [28]. In the current study, increasing the space between treatments decreased males captured with pheromone lures in proportion to those captured with females in pistachio (Table 3, percent of female baited); consistent with this previous study. In almonds, though, increased space between traps had the opposite effect; it increased the proportion of males captured with lures. These data suggest that the synthetic pheromone lure performs more similarly to females in almonds than in pistachios. If this difference between crops in response to pheromones is real, then it could possibly be due to the influence of background volatiles on the response to pheromone [33,34]. Studies on the codling moth identified a host-related volatile, ethyl (*E*,*Z*)-2,4,-decadienoate, that enhances the male response to sex pheromones [35]. The difference in the density of plantings and canopies between almonds and pistachios is another possible factor in interactions of the males with pheromone plumes.

It is also possible that an effect of population density on the relative attractiveness of calling females and synthetic lures was an important (or even principal) factor in the apparent crop differences observed in this study. This interpretation is supported by Days 26 through 40 of Experiment 1 (Figure 1). This experiment was conducted in almonds at a time of higher abundance, and during this period, traps baited with females significantly outperformed traps baited with synthetic pheromone lures. It is possible that the preference of males for calling females over the synthetic pheromone formulation is density dependent. This density dependence suggests that the lures are capable of attracting males from a considerable range, but that the males prefer plumes of authentic pheromone from calling females. Previous studies have demonstrated that free-flying calling females compete with static traps baited with calling females or synthetic pheromone [36,37,38]. Such an effect would presumably be greater if males prefer the females over the artificial pheromone source. More studies are needed to determine factors involved in the difference in the response of navel orangeworm males to traps baited with the pheromone lure *vs*. female-baited traps and to determine how best to use this lure for pest management in the crops in which navel orangeworm is a pest.

## 4. Conclusions

A persistent pheromone lure for the navel orangeworm has been developed and is available for the first time for this economically important species. This lure attracts navel orangeworm and provides reliable monitoring for at least 40 days. Fluctuations in males captured baited with the pheromone lure generally correlate significantly with captures in female-baited traps, suggesting that this pheromone lure will be suitable for timing treatment and other pest management events. The experiments reported here, however, suggest that this lure attracts males less efficiently compared to calling females and that male response to this lure may differ between almonds and pistachios and/or with population density. These observations suggest that, pending further studies and greater experience, particular caution should be taken if trapping data with this lure is used to infer abundance or to compare between crops. These issues notwithstanding, this persistent lure represents a marked improvement over the egg traps previously used for monitoring.
